# Selectively increasing of polyunsaturated (18:2) and monounsaturated (18:1) fatty acids in *Jatropha curcas* seed oil by crystallization using D-optimal design

**DOI:** 10.1186/1752-153X-6-65

**Published:** 2012-07-02

**Authors:** Jumat Salimon, Bashar Mudhaffar Abdullah, Nadia Salih

**Affiliations:** 1 School of Chemical Sciences and Food Technology, Faculty of Science and Technology, Universiti Kebangsaan Malaysia, 43600, Bangi, Selangor, Malaysia

**Keywords:** D-optimal design, Optimization, Polyunsaturated, Monounsaturated, Linoleic acid, Oleic Acid

## Abstract

**Background:**

This study was done to obtain concentrated polyunsaturated fatty acid (PUFA) linoleic acid (LA; 18:2) and monounsaturated fatty acid (MUFA) oleic acid (OA; 18:1) from *Jatropha curcas* seed oil by urea complexation. Urea complexation is a method used by researchers to separate fatty acids (FAs) based on their molecular structure. Effects the ratio of urea-to-FAs, crystallization temperature and crystallization time on the final products of urea complexation were examined. D-optimal Design was employed to study the significance of these factors and the optimum conditions for the technique were predicted and verified.

**Results:**

Optimum conditions of the experiment to obtain maximum concentration of LA were predicted at urea-to-FAs ratio (w/w) of 5:1, crystallization temperature of −10°C and 24 h of crystallization time. Under these conditions, the final non-urea complex fraction (NUCF) was predicted to contain 92.81% of LA with the NUCF yield of 7.8%. The highest percentage of OA (56.01%) was observed for samples treated with 3:1 urea-to-FAs ratio (w/w) at 10°C for 16 h. The lowest percentage of LA (8.13%) was incorporated into urea complex fraction (UCF) with 1:1 urea-to-FAs ratio (w/w) at 10°C for 8 h.

**Conclusions:**

The separation of PUFA (LA) and MUFA (OA) described here. Experimental variables should be carefully controlled in order to recover a maximum content of PUFA and MUFA of interest with reasonable yield% with a desirable purity of fatty acid of interest.

## Background

Linoleic acid (LA) [also called *cis,cis*,-9,12-octadecadienoic acid] is an example of a polyunsaturated fatty acid (PUFA), due to the presence of two carbon double bonds. The high content of LA makes *Jatropha curcas* seed oil very important for industry use. LA can be used in protective coatings, plastics, surfactant, dispersants, biolubricant, and a variety of synthetic and in the preparations of other long chain compounds. The high content of LA in seed oil of *J. curcas* is very important to the production of oleo-chemicals [1]. Oleic acid (OA) [also called (9z)- octadec-9-enoic acid] is an example of a monounsaturated fatty acid (MUFA). A small amount of OA is used in the pharmaceutical industry, as an emulsifying agent in aerosol products [2].

There are several methods which can be used to obtain polyunsaturated fatty acids (PUFA) including freezing crystallization, urea complexation, molecular distillation, supercritical fluid extraction, silver ion complexation and lipase concentration [3] as well as high-performance liquid chromatography [4]. The most economic and most efficient technique to obtain LA in the form of fatty acids (FAs) is urea complex fractionation. This is a well-established technique for the elimination of saturated fatty acids (SFAs) and MUFA [5].

Urea complexation has the advantage that the complex crystals are extremely stable, and filtration is not carried out at low temperatures which is required for solvent crystallization of FAs. This method is preferred by many researchers because complexation depends upon configuration of the FAs moieties due to the presence of multiple double bonds, rather than pure physical properties such as melting point or solubility [5,6]. The SFAs and MUFA easily form complexes with urea and crystallize out at cooling during urea complex fraction (UCF). These complexes can subsequently be removed by filtration. The liquid or non-urea complex fraction (NUCF) is enriched with PUFA and the crystals formed or UCF consists of SFAs and MUFA.

In this study, urea complex fractionation of a mixture of FAs of Malaysian *J. curcas* seed oil was carried out to obtain concentrated PUFA. The effects of urea-to-FAs ratio, crystallization temperature and crystallization time to the yield% of NUCF (*Y*_*1*_), yield% of UCF (*Y*_*5*_), percentage MUFA (OA) (*Y*_*3*_ and *Y*_*7*_) and percentage PUFA (LA) (*Y*_*4*_ and *Y*_*5*_) in NUCF and UCF were systematically studied.

## Results and discussion

### Non-urea complex fraction (NUCF)

The original fatty acids (FAs) mixture was composed of 13.19% palmitic (16:0), 6.37% stearic (18:0), 43.33% oleic (18:1) and 36.71% linoleic (18:2) acid. Average molecular weight of the FAs was 203.36 as obtained from saponification test of the original oil. The results compared well with those of [7]. The PUFA (LA) concentrate was prepared by urea complex fractionation following the technique of [8], using the FAs that was previously obtained. The purpose of this procedure was to obtain a PUFA concentrate enriched in LA and simultaneously, maintain the highest yield% of LA. The crystallization process with urea preferentially selects SFAs and MUFA, and the tendency of FAs to combine with urea decreases with increasing chain lengths [9].

In this study, variations of factors that affect the urea complex fractionation such as the ratio of urea-to-FAs (w/w), crystallization temperature (°C) and crystallization time (h) were examined to obtain optimum conditions using the response surface method D-optimal design. Table 1 shows data obtained from the experiment on FAs composition in the NUCF of all the samples. Results showed that the percentage of LA has increased from 36.71% to as much as 92.81% while SFAs (0.33%) has been reduced considerably compared to the initial FAs mixture. In samples with high ratio of urea-to-FAs, the elimination of SFAs was near completion in NUCF. However, total removal of oleic, palmitic and stearic acids by urea complexation may be impossible because some of the SFAs do not form complexes with urea during crystallization [10]. These results demonstrate that oleic, palmitic and stearic acids have more tendencies to form urea adducts than LA.

**Table 1:** **D-optimal design arrangement and responses for non-urea-complexed fraction (NUCF) of *Jatropha curcas *seed oil**

	**Variables levels**			**Responses, *Y***					
**Run no.**	**Urea-FAs**^**a **^**(*X***_***1***_**)**	**Temp.**^**b **^**(*X***_***2***_**)**	**Time**^**c **^**(*X***_***3***_**)**	***Y***_***1***_**, Yield (%)**	**C16:0 (%)**	**C18:0 (%)**	***Y***_***2***_**, SFAs (C16:0+C18:0) (%)**	***Y***_***3***_**, MUFA (C18:1) (%)**	***Y***_***4***_**, LA (C18:2) (%)**
**1**	1	10	8	49	1.44	-	1.44	36.50	58.23
**2**	3	0	24	22.2	0.56	0.26	0.82	13.25	85.16
**3**	2	0	16	34.9	0.44	-	0.44	28.87	69.41
**4**	3	-10	8	32.3	0.49	-	0.49	20.90	77.74
**5**	5	10	24	7.7	0.43	-	0.43	9.37	88.60
**6**	5	-10	24	7.8	0.33	-	0.33	5.73	92.81
**7**	1	10	24	50.6	3.25	0.30	3.55	39.67	54.91
**8**	1	-10	24	34.1	0.58	-	0.58	34.64	61.46
**9**	5	10	8	8.8	1.23	-	1.23	9.94	87.82
**10**	5	0	16	6.2	0.97	-	0.97	8.95	89.19
**11**	1	-10	16	48.1	1.17	-	1.17	35.55	59.85
**12**	1	0	8	31.3	2.79	-	2.79	39.58	54.63
**13**	5	10	24	4.1	0.94	0.28	1.23	6.31	92.14
**14**	3	10	16	31.6	0.19	-	0.19	20.09	78.42
**15**	1	10	8	45.6	3.65	0.33	3.99	40.49	52.53
**16**	5	-10	8	6.6	0.89	-	0.89	9.10	88.92
**17**	5	0	8	20.5	0.34	-	0.34	10.30	88.12
**18**	1	10	16	49.7	1.17	-	1.17	41.30	54.87

Notes: C16:0, palmitic acid; C18:0, stearic acid; C18:1, oleic acid; C18:1, linoleic acid; SFAs, saturated fatty acids; MUFA, monounsaturated fatty acid; LA, linoleic acid;

^a^ urea-to-FAs ratio (w/w).

^b^ crystallization temperature (Â°C).

^c^ crystallization time (h).

The LA% derived from the NUCF phase was relatively high, and some even greater than 90% under certain experimental conditions (Table 1). This showed that the experimental conditions were suitable for the preparation of high purity LA. However, it is difficult to completely remove all the SFAs and MUFA to obtain 100% purity of PUFA in the concentrate. [11] reported that complete removal of SFAs and MUFA by urea complexation may be impossible since some of the SFAs do not bind with urea during crystallization.

The quadratic regression coefficient obtained by employing a least squares method to predict quadratic polynomial models for the yield% of NUCF (*Y*_*1*_), percentage of SFAs (palmitic and stearic acids) (*Y*_*2*_), percentage of MUFA (OA) (*Y*_*3*_) and percentage of PUFA (LA) (*Y*_*4*_) in NUCF are given in Tables 2, 3, 4 and 5 respectively.

**Table 2:** **Regression coefficients of the predicted quadratic polynomial model for response variables (yield% of NUCF) in urea inclusion fractionation experiment of *J. curcas *seed oil**

**Variables**	**Coefficients (ß), yield % of NUCF (*Y***_***1***_**)**	***T***	***P***	**Notability**
Intercept	27.97	11.71	0.0010	***
Linear				
*X*_*1*_	-16.60	73.24	0.0001	***
*X*_*2*_	1.85	0.88	0.3767	
*X*_*3*_	-1.60	0.66	0.4405	
Quadratic				
*X*_*11*_	-2.39	0.31	0.5935	
*X*_*22*_	3.88	1.09	0.3263	
*X*_*33*_	-3.43	0.84	0.3849	
Interaction				
*X*_*12*_	-2.44	1.28	0.2904	
*X*_*13*_	-2.13	1.02	0.3428	
*X*_*23*_	0.69	0.093	0.7682	
*R*^*2*^	0.92			

Notes: ** *P <* 0.05; *** *P <* 0.01. T: F test value.

See Table 1 for a description of the abbreviations.

**Table 3:** **Regression coefficients of the predicted quadratic polynomial model for response variables (SFAs%) in urea inclusion fractionation experiment of *J. curcas *seed oil**

**Variables**	**Coefficients (ß), SFAs% (*Y***_***2***_**)**	***T***	***P***	**Notability**
Intercept	0.087	1.84	0.2005	
Linear				
*X*_*1*_	-0.69	6.65	0.0327	**
*X*_*2*_	0.40	2.12	0.1831	
*X*_*3*_	-0.12	0.21	0.6600	
Quadratic				
*X*_*11*_	0.92	2.44	0.1572	
*X*_*22*_	-0.31	0.36	0.5672	
*X*_*33*_	0.72	1.98	0.1972	
Interaction				
*X*_*12*_	-0.24	0.68	0.4342	
*X*_*13*_	0.041	0.020	0.8914	
*X*_*23*_	0.26	0.67	0.4370	
*R*^*2*^	0.67			

Notes: ** *P <* 0.05; *** *P <* 0.01. T: F test value

See Table 1 for a description of the abbreviations

**Table 4:** **Regression coefficients of the predicted quadratic polynomial model for response variables (MUFA (OA%)) in urea inclusion fractionation experiment of *J. curcas *seed oil**

**Variables**	**Coefficients (ß), MUFA (OA%) (*Y***_***3***_**)**	***T***	***P***	**Notability**
Intercept	18.77	81.33	0.0001	***
Linear				
*X*_*1*_	-14.76	577.20	0.0001	***
*X*_*2*_	1.04	2.80	0.1330	
*X*_*3*_	-1.59	6.53	0.0339	**
Quadratic				
*X*_*11*_	5.19	14.55	0.0051	***
*X*_*22*_	0.29	0.061	0.8115	
*X*_*33*_	-1.32	1.25	0.2951	
Interaction				
*X*_*12*_	-0.49	0.52	0.4906	
*X*_*13*_	-0.39	0.34	0.5741	
*X*_*23*_	1.08	2.26	0.1714	
*R*^*2*^	0.98			

Notes: ** *P <* 0.05; *** *P <* 0.01. T: F test value

See Table 1 for a description of the abbreviations

**Table 5:** **Regression coefficients of the predicted quadratic polynomial model for response variables (PUFA (LA%)) in urea inclusion fractionation experiment of *J. curcas *seed oil**

**Variables**	**Coefficients (ß), PUFA (LA%) (*Y***_***4***_)	***T***	***P***	**Notability**
Intercept	80.30	91.62	0.0001	***
Linear				
*X*_*1*_	16.37	643.86	0.0001	***
*X*_*2*_	-1.30	3.95	0.0820	
*X*_*3*_	1.91	8.49	0.0195	**
Quadratic				
*X*_*11*_	-7.14	24.89	0.0011	***
*X*_*22*_	-0.22	0.032	0.8622	
*X*_*33*_	0.65	0.28	0.6133	
Interaction				
*X*_*12*_	0.57	0.64	0.4466	
*X*_*13*_	0.18	0.065	0.8056	
*X*_*23*_	-1.10	2.13	0.1827	
*R*^*2*^	0.99			

Notes: ** *P <* 0.05; *** *P <* 0.01. T: F test value

See Table 1 for a description of the abbreviations

Examination of these coefficients with a *T-*test shows that the percentage yield of NUCF (*Y*_*1*_), percentage of MUFA (OA) (*Y*_*3*_), percentage of PUFA (LA) (*Y*_*4*_), the linear term of urea-to-FAs ratio (*X*_*1*_) and quadratic term of urea-to-FAs were highly significant (*p* < 0.01), while the percentage of SFAs (*Y*_*2*_), the linear term was significant at *p* < 0.05. Lastly, linear term of crystallization time (*X*_*3*_) for the percentage of PUFA (LA) (*Y*_*4*_) and percentage of MUFA (LA) (*Y*_*3*_) in the concentrate were significant at *p* < 0.05.

The results suggest that the linear effect of urea-to-FAs ratio and crystallization time are the primary determining factors for FAs separation by urea complexation. [10] concluded that these two variables significantly influenced the results of their urea complexation study. Crystallization time was found to be the insignificant factor (*P >* 0.05). This finding is in agreement with the results reported by other researchers [5,6,10].

The coefficients of independent variables (urea-to-FAs ratio; *X*_*1*_, crystallization temperature; *X*_*2*_ and crystallization time; *X*_*3*_) determined for the quadratic polynomial models are given in Tables 2, 3, 4 and 5. Table 2 lists the yield% of NUCF (*Y*_*1*_); Table 3 the percentage of SFAs (palmitic and stearic acids) (*Y*_*2*_); Table 4 the percentage of MUFA (OA) (*Y*_*3*_) and Table 5 percentage of PUFA (LA) (*Y*_*4*_) in NUCF are given below:

(1) $$\begin{array}{ll} Y_{1}= & +27.97-16.60X_{1}+1.85X_{2}-1.60X_{3}\\ & -2.39X_{1}{}^{2}+3.88X_{2}{}^{2}-3.43X_{3}{}^{2}\\ & -2.44X_{1}X_{2}-2.13X_{1}X_{3}+0.69X_{2}X_{3} \end{array}$$

(2) $$\begin{array}{ll} Y_{2}= & +0.087-0.69X_{1}+0.40X_{2}-0.12X_{3}\\ & +0.92X_{1}{}^{2}-0.031X_{2}{}^{2}+0.72X_{3}{}^{2}\\ & -0.24X_{1}X_{2}+0.041X_{1}X_{3}+0.26X_{2}X_{3} \end{array}$$

(3) $$\begin{array}{ll} Y_{3}= & +18.77-14.76X_{1}+1.04X_{2}-1.59X_{3}\\ & +5.19X_{1}{}^{2}+0.29X_{2}{}^{2}-1.32X_{3}{}^{2}\\ & -0.49X_{1}X_{2}-0.39X_{1}X_{3}+1.08X_{2}X_{3} \end{array}$$

(4) $$\begin{array}{ll} Y_{4}= & +80.30+16.37X_{1}-1.30X_{2}+1.91X3\\ & -7.14X_{1}{}^{2}-0.22X_{2}{}^{2}+0.65X_{3}{}^{2}\\ & +0.57X_{1}X_{2}+0.18X_{1}X_{3}-1.10X_{2}X_{3} \end{array}$$

The *F*-value for the lack-of-fit for all the responses (Table 6) showed that the lack of fit is not significant (*p* > 0.05) relative to the pure error. This indicates that all the models predicted for the responses were adequate. The regression coefficients (*R*^*2*^) of the yield% of NUCF (*Y*_*1*_), percentage of SFAs (palmitic and stearic acids) (*Y*_*2*_), percentage of MUFA (OA) (*Y*_*3*_) and percentage of PUFA (LA) (*Y*_*4*_) in NUCF were 0.92, 0.67, 0.98 and 0.99, respectively (Table 2, 3, 4 and 5, respectively). These indicate that the generated models adequately explained the data variation and represented the actual relationships among the reaction parameters.

**Table 6:** Analysis of variance (ANOVA) for all the responses of NUCF

	**Source**	***Df***	**Sum of squares**	**Mean square**	***F* value**	***P***	
*Y*_*1*_	Model	3	4484.17	1494.72	37.83	< 0.0001	Significant
	Residual	14	553.23	39.52			
	lack-of-fit	12	540.97	45.08	7.35	0.1258	Not significant
	Pure error	2	12.26	6.13			
*Y*_*2*_	Model	3	8.44	2.81	3.20	0.0560	Not significant
	Residual	14	12.29	0.88			
	lack-of-fit	12	8.73	0.73	0.41	0.8720	Not significant
	Pure error	2	3.57	1.78			
*Y*_*3*_	Model	9	3259.79	362.20	81.33	< 0.0001	Significant
	Residual	8	35.63	4.45			
	lack-of-fit	6	23.03	3.84	0.61	0.7298	Not significant
	Pure error	2	12.59	6.30			
*Y*_*4*_	Model	9	4051.33	450.15	91.62	< 0.0001	Significant
	Residual	8	39.31	4.91			
	lack-of-fit	6	16.80	2.80	0.25	0.9219	Not significant
	Pure error	2	22.50	11.25			

**Figure 1:**
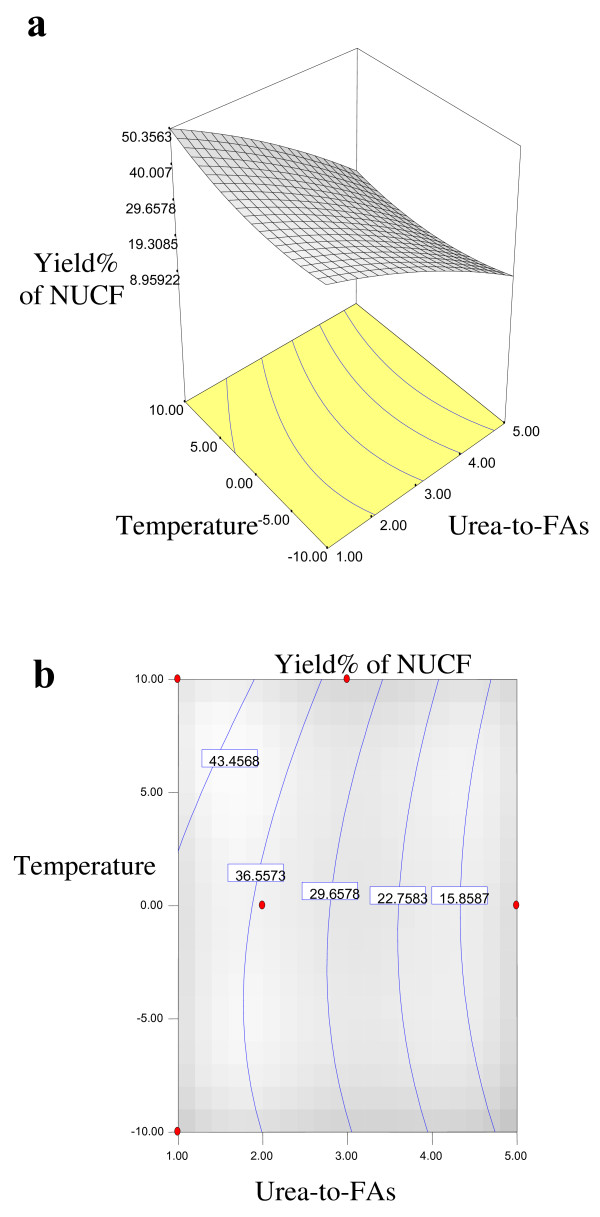
**Response surface (a) and contour plots (b) for the effect of the urea-to-FAs ratio (*X***_***1***_**, w/w) and crystallization temperature (*X***_***2***_**, °C) on the yield% (*Y***_***1***_**) of NUCF.**

**Figure 2:**
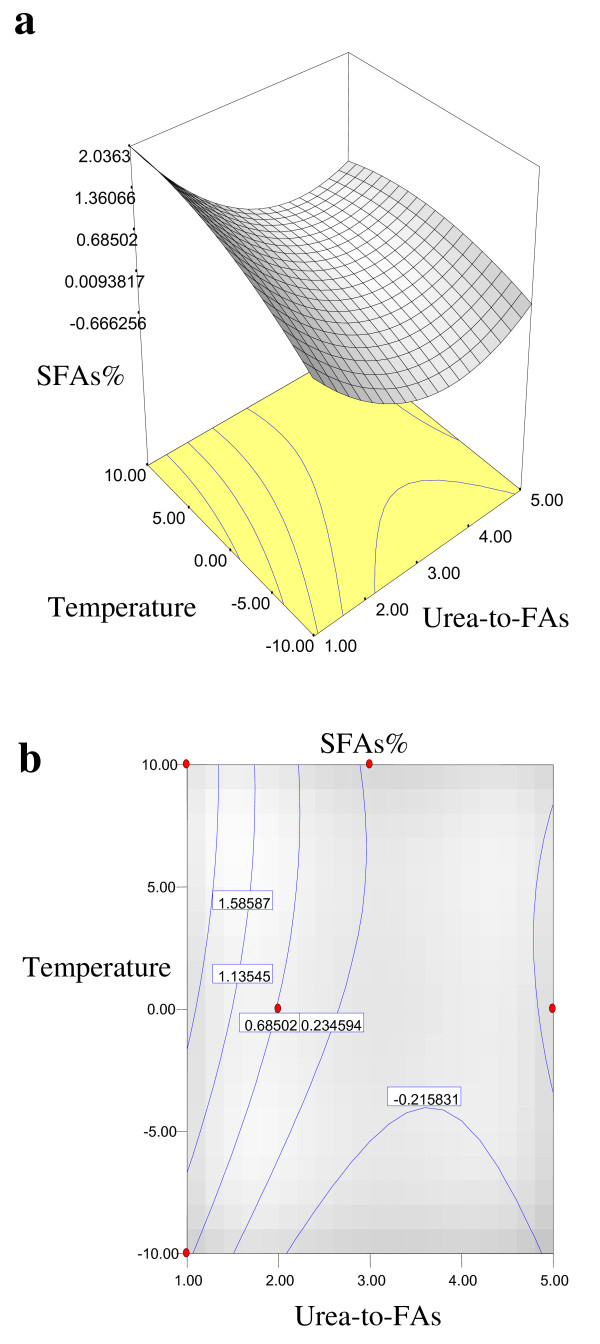
**Response surface (a0 and contour plots (b) for the effect of the urea-to-FAs ratio (*X***_***1***_**, w/w) and crystallization temperature (*X***_***2***_**, °C) on the SFAs% (*Y***_***2***_**) of NUCF.**

**Figure 3:**
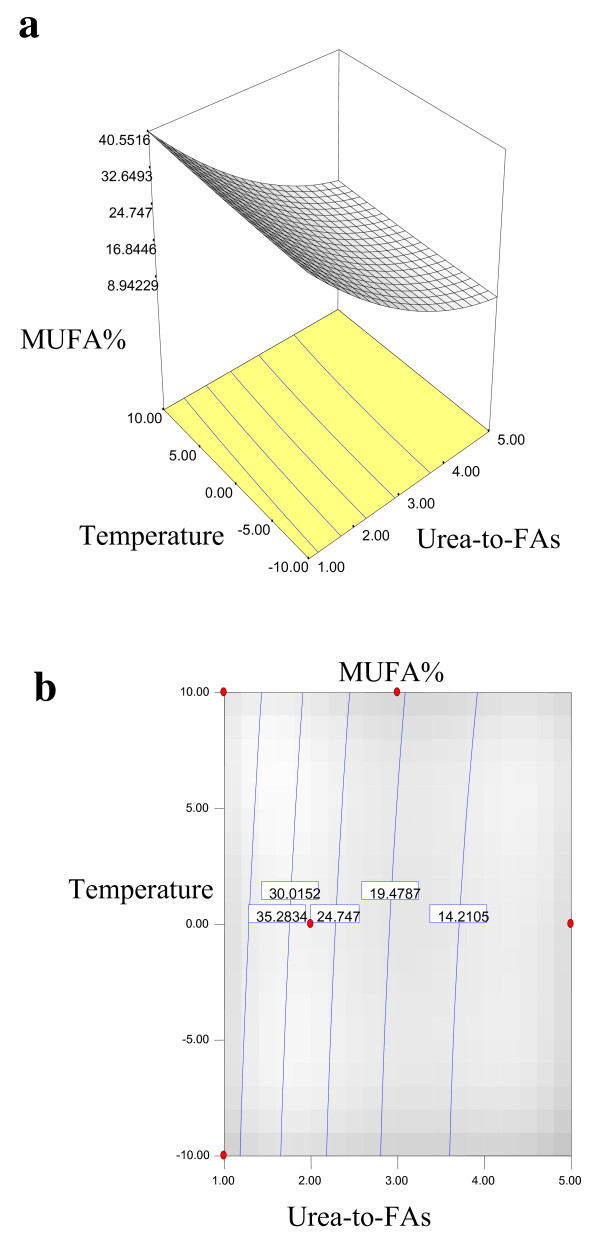
**Response surface (a) and contour plots (b) for the effect of the urea-to-FAs ratio (*X***_***1***_**, w/w) and crystallization temperature (*X***_***2***_**, °C) on the MUFA (OA%) (*Y***_***3***_**) of NUCF.**

**Figure 4:**
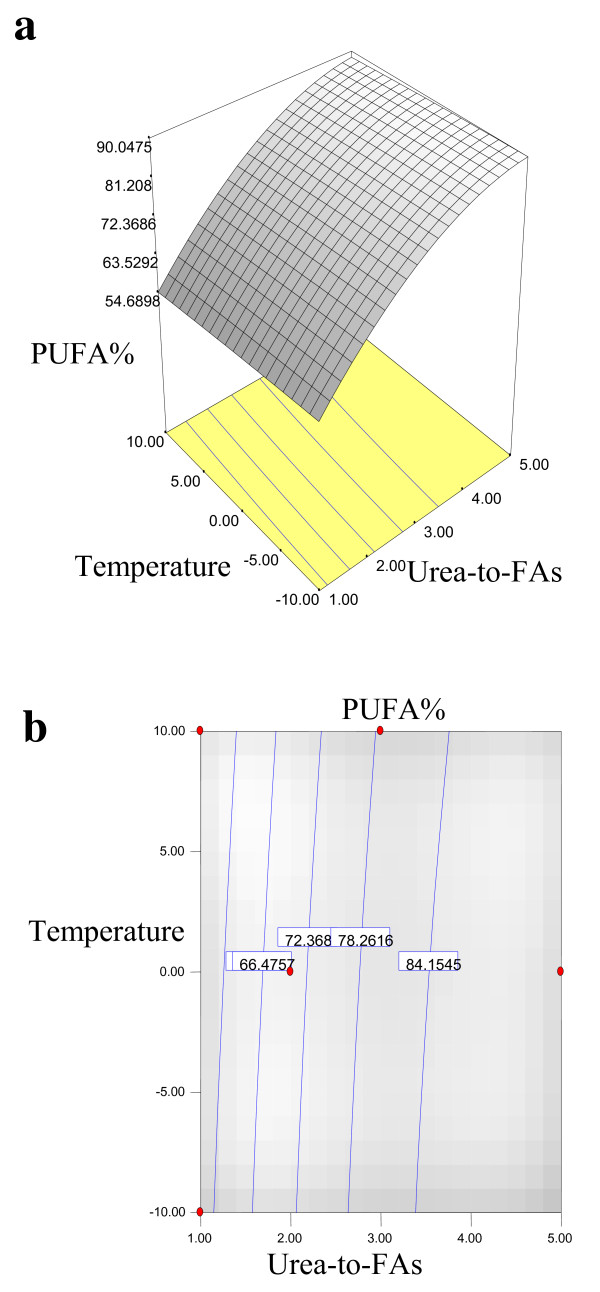
**Response surface (a) and contour plots (b) for the effect of the urea-to-FAs ratio (*X***_***1***_**, w/w) and crystallization temperature (*X***_***2***_**, °C) on the PUFA (LA%) (*Y***_***4***_**) of NUCF.**

Equations 1, 2, 3 and 4 showed that the yield% of NUCF (*Y*_*1*_), percentage of SFAs (palmitic and stearic acids) (*Y*_*2*_), percentage of MUFA (OA) (*Y*_*3*_) and percentage of PUFA (LA) (*Y*_*4*_) in NUCF have a complex relationship with independent variables that encompass both first- and second-order polynomials. The relationships between independent and dependent variables are shown in the three-dimensional representation as response surfaces. The response surfaces for the yield% of NUCF (*Y*_*1*_), percentage of SFAs (palmitic and stearic acids) (*Y*_*2*_), percentage of MUFA (OA) (*Y*_*3*_) and percentage of PUFA (LA) (*Y*_*4*_) in NUCF in the concentrates are given in Figures 1, 2, 3 and 4, respectively. The contour plots (Figures 1b, 2b, 3b and 4b) show the combination of levels of the urea-to-FAs ratio that can afford the same level of the yield% of NUCF (*Y*_*1*_), percentage of SFAs (palmitic and stearic acids) (*Y*_*2*_), percentage of MUFA (OA) (*Y*_*3*_) and percentage of PUFA (LA) (*Y*_*4*_) in NUCF.

Figures 1, 2, 3 and 4 show increasing amount of urea and decreasing crystallization temperature which led to reduction of percentage of SFAs and MUFA (OA) in liquid NUCF. The content of PUFA (LA) in the liquid fraction would also be enriched under these conditions (Figure 4). The relationships between the parameters and FAs percentages were linear or almost linear. High concentration of PUFA (LA) could be obtained by using high ratio of urea-to-FAs at low temperatures. However, this could also reduce the yield% of liquid NUCF in the final product as more LA would be lost into urea adducts. Experimental variables should therefore be carefully controlled in order to recover a maximum content of PUFA (LA) of interest with reasonable yield% [5].

Straight-chained molecules such as SFAs readily formed stable adduct with urea. SFAs formed complexes more readily than MUFA. MUFA formed more readily inclusion compounds than PUFA (LA). Similar complexation tendency patterns were also obtained by [12]. The addition of more urea could reduce the SFAs percentage in NUCF to a minimum level; it however results in indiscriminate FAs complexation and thus reducing the amount of MUFA (OA) and PUFA (LA). A lower urea-to-FAs ratio prevented indiscriminate FAs complexation. Lower crystallization temperature can facilitate formation of more stable urea adducts, that would reduce SFAs in NUCF. Longer periods of crystallization time would allow the crystals to further stabilize. However the parameters must be set at a level to achieve an acceptable yield% of product with high purity. Higher purity of PUFA (LA) will always give lower yield of NUCF.

**Figure 5:**
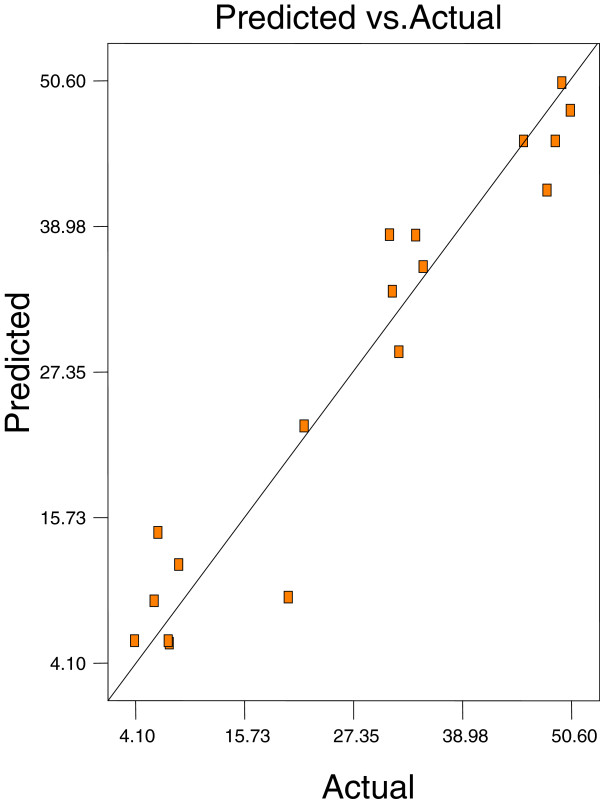
**Predicated vs. actual plot of *Y***_***1***_.

**Figure 6:**
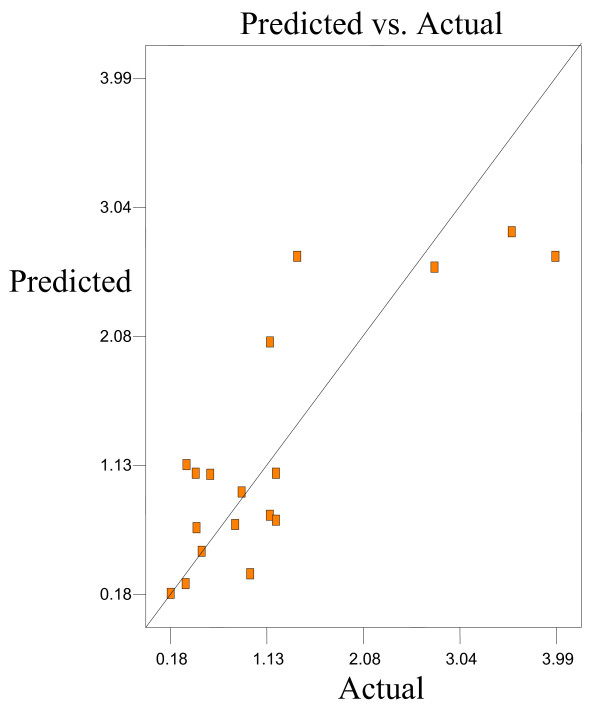
**Predicated vs. actual plot of *Y***_***2***_.

**Figure 7:**
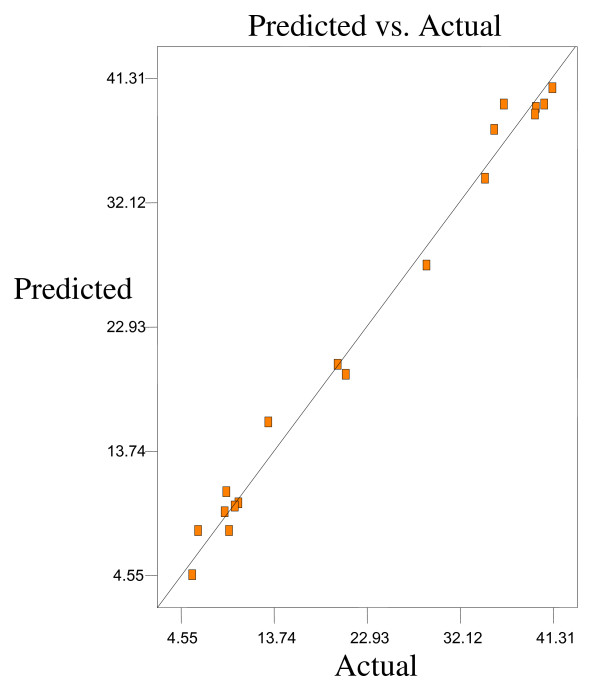
**Predicated vs. actual plot of *Y***_***3***_.

**Figure 8:**
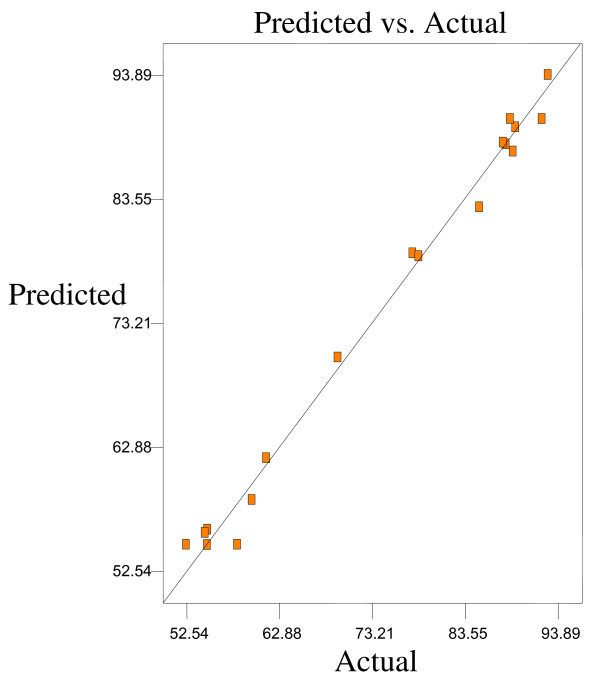
**Predicated vs. actual plot of *Y***_***4***_.

Optimum conditions using D-optimal design to obtain maximum concentration of PUFA (LA) and minimum concentration of both SFAs (palmitic and stearic acids) and MUFA (OA) were predicted at a urea-to-FAs ratio (w/w) of 5:1, crystallization temperature of −10°C and 24 h of crystallization time. The final NUCF was predicted to contain 0.33% of SFAs (palmitic and stearic acids), 5.73% of MUFA (OA) and 92.81% of PUFA (LA) with the NUCF yield of 7.8%. The observed value was reasonably close to the predicted value as shown in Figures 5, 6, 7 and 8.

### Urea complex fraction (UCF)

The crystallization process with urea complex fraction (UCF) selects SFAs (palmitic and stearic acids) and MUFA (OA), simultaneously maintain the highest yield% of SFAs and MUFA (OA). The new tendency of FAs to combine with urea decreases with increasing UFAs [9]. Table 7 shows the FAs composition in the UCF. The SFAs and MUFA (OA) percentage were significantly higher compared to the starting material while PUFA (LA) was lower in all the samples. The highest percentage of SFAs (44.27%) was observed for sample treated with 1:1 urea-to-FAs ratio (w/w) at 10°C for 16 h.

**Table 7:** **D-optimal design arrangement and responses for urea-complexed fraction (UCF) of *Jatropha curcas *seed oil**

	**Variables levels**			**Responses, *Y***					
**Run no.**	**Urea-FAs**^**a **^**(*X***_***1***_**)**	**Temp.**^**b **^**(*X***_***2***_**)**	**Time**^**c **^**(*X***_***3***_**)**	***Y***_***5***_**, Yield (%)**	**C16:0 (%)**	**C18:0 (%)**	***Y***_***6***_**, SFAs (C16:0+C18:0) (%)**	***Y***_***7***_**, MUFA (C18:1) (%)**	***Y***_***8***_**, LA (C18:2) (%)**
1	1	10	8	50.7	25.42	13.97	39.39	48.10	8.13
2	3	0	24	78.6	17.09	9.27	26.36	53.15	19.94
3	2	0	16	64.9	22.62	21.50	44.13	44.89	10.23
4	3	−10	8	67.4	19.34	10.28	29.62	54.11	15.47
5	5	10	24	92.1	14.14	7.36	21.50	45.07	29.34
6	5	−10	24	92.0	20.59	12.43	33.02	55.41	8.37
7	1	10	24	48.7	25.06	14.57	39.64	45.69	11.50
8	1	−10	24	65.7	23.06	12.24	35.31	45.63	15.74
9	5	10	8	91.0	13.52	8.11	21.63	42.61	31.45
10	5	0	16	93.5	14.47	8.91	23.39	44.44	28.68
11	1	−10	16	51.3	19.76	10.39	30.16	45.28	20.83
12	1	0	8	68.8	20.37	11.84	32.21	43.89	20.10
13	5	10	24	95.7	13.40	6.52	19.92	42.38	35.81
14	3	10	16	68.3	20.37	11.08	31.46	56.01	12.10
15	1	10	8	54.3	23.34	13.49	36.84	45.28	14.61
16	5	−10	8	93.2	13.68	8.33	22.01	42.89	30.77
17	5	0	8	79.2	16.61	8.94	25.56	53.00	20.87
18	1	10	16	49.8	28.38	15.89	44.27	43.04	9.46

Notes: C16:0, palmitic acid; C18:0, stearic acid; C18:1, oleic acid; C18:2, linoleic acid; SFAs, saturated fatty acids; MUFA, monounsaturated fatty acid; LA, linoleic acid;

^a^ urea-to-FAs ratio (w/w).

^b^ crystallization temperature (Â°C).

^c^ crystallization time (h).

The highest percentage of MUFA (OA) (56.01%) was observed for samples treated with 3:1 urea-to-FAs ratio (w/w) at 10°C for 16 h, while the lowest percentage of PUFA (LA) (8.13%) was incorporated into the urea complex with 1:1 urea-to-FAs ratio (w/w) at 10°C for 8 h. Inclusion of more PUFA (LA) into UCF reduced the percentage of SFAs and MUFA (OA) in the samples. The process may be not suitable for industrial uses because this method cannot employ high purity SFAs (palmitic and stearic acids) and MUFA (OA).

The quadratic regression coefficient obtained by employing a least squares method to predict quadratic polynomial models for the yield% of solid UCF (*Y*_*5*_), percentage SFAs (palmitic and stearic acids) (*Y*_*6*_), percentage MUFA (OA) (*Y*_*7*_) and percentage PUFA (LA) (*Y*_*8*_) are given in Tables 8, 9, 10 and 11 respectively.

**Table 8:** **Regression coefficients of the predicted quadratic polynomial model for response variables (yield% of UCF) in urea inclusion fractionation experiment of *J. curcas *seed oil**

**Variables**	**Coefficients (ß), yield % of UCF (*Y***_***5***_**)**	***T***	***P***	**Notability**
Intercept	72.14	11.29	0.0012	***
Linear				
*X*_*1*_	16.64	70.27	0.0001	***
*X*_*2*_	-1.83	0.82	0.3911	
*X*_*3*_	1.65	0.67	0.4370	
Quadratic				
*X*_*11*_	1.97	0.20	0.6662	
*X*_*22*_	-4.18	1.21	0.3038	
*X*_*33*_	3.74	0.96	0.3568	
Interaction				
*X*_*12*_	2.46	1.25	0.2965	
*X*_*13*_	2.21	1.04	0.3374	
*X*_*23*_	-0.81	0.12	0.7362	
*R*^*2*^	0.92			

Notes: ** *P <* 0.05; *** *P <* 0.01. T: F test value.

See Table 7 for a description of the abbreviations.

**Table 9:** **Regression coefficients of the predicted quadratic polynomial model for response variables (SFAs%) in urea inclusion fractionation experiment of *J. curcas *seed oil**

**Variables**	**Coefficients (ß), SFA% (*Y***_***6***_**)**	***T***	***P***	**Notability**
Intercept	33.80	4.51	0.0226	**
Linear				
*X*_*1*_	-5.43	16.17	0.0038	***
*X*_*2*_	0.38	0.076	0.7898	
*X*_*3*_	1.49	1.17	0.3101	
Quadratic				
*X*_*11*_	-1.22	0.17	0.6943	
*X*_*22*_	-0.053	4.140E-004	0.9843	
*X*_*33*_	-3.06	1.39	0.2719	
Interaction				
*X*_*12*_	-3.96	6.99	0.0295	**
*X*_*13*_	-0.41	0.078	0.7868	
*X*_*23*_	-1.78	1.28	0.2913	
*R*^*2*^	0.83			

Notes: ** *P <* 0.05; *** *P <* 0.01. T: F test value.

See Table 7 for a description of the abbreviations.

**Table 10:** **Regression coefficients of the predicted quadratic polynomial model for response variables (MUFA (OA%)) in urea inclusion fractionation experiment of *J. curcas *seed oil**

**Variables**	**Coefficients (ß), MUFA (OA%) (*Y***_***7***_**)**	***T***	***P***	**Notability**
Intercept	52.35	1.43	0.3118	
Linear				
*X*_*1*_	1.40	1.26	0.2940	
*X*_*2*_	-1.07	0.72	0.4218	
*X*_*3*_	0.78	0.38	0.5571	
Quadratic				
*X*_*11*_	-8.33	9.08	0.0167	**
*X*_*22*_	0.71	0.089	0.7728	
*X*_*33*_	1.58	0.43	0.5281	
Interaction				
*X*_*12*_	-1.68	1.47	0.2604	
*X*_*13*_	0.39	0.082	0.7821	
*X*_*23*_	-1.89	1.68	0.2315	
*R*^*2*^	0.61			

Notes: ** *P <* 0.05; *** *P* < 0.01. T: F test value.

See Table 7 for a description of the abbreviations.

**Table 11:** **Regression coefficients of the predicted quadratic polynomial model for response variables (PUFA (LA%)) in urea inclusion fractionation experiment of *J. curcas *seed oil**

**Variables**	**Coefficients (ß), PUFA (LA%) (*Y***_***8***_**)**	***T***	***P***	**Notability**
Intercept	13.73	3.77	0.0375	**
Linear				
*X*_*1*_	4.16	6.42	0.0350	**
*X*_*2*_	0.83	0.25	0.6321	
*X*_*3*_	-2.07	1.54	0.2500	
Quadratic				
*X*_*11*_	6.88	3.59	0.0949	
*X*_*22*_	-1.44	0.21	0.6592	
*X*_*33*_	1.55	0.24	0.6360	
Interaction				
*X*_*12*_	5.61	9.49	0.0151	**
*X*_*13*_	-0.15	6.701E-003	0.9368	
*X*_*23*_	3.74	3.79	0.0874	
*R*^*2*^	0.80			

Notes: ** *P* < 0.05; *** *P* < 0.01. T: F test value.

See Table 7 for a description of the abbreviations.

Linear term of urea-to-FAs ratio was highly significant (*p* < 0.01) for the yield% of UCF (*Y*_*5*_) and percentage SFAs (*Y*_*6*_), while the linear term of urea-to-FAs was significant (*P* < 0.05) for the percentage PUFA (LA) (*Y*_*8*_). The interaction between urea-to-FAs ratio and crystallization temperature were significant (*p* < 0.05) for the percentage PUFA (LA) (*Y*_*8*_) and the percentage SFAs (*Y*_*6*_). Quadratic term of urea-to-FAs ratio was also significant (*p* < 0.05) for the percentage MUFA (OA) (*Y*_*7*_).

The coefficients of the independent variables (urea-to-FAs ratio; *X*_*1*_, crystallization temperature; *X*_*2*_ and crystallization time; *X*_*3*_) determined for the quadratic polynomial models are lists in Tables 8, 9, 10 and 11 respectively. Table 8 lists the yield% of solid UCF (*Y*_*5*_), Table 9 lists the percentage SFAs (palmitic and stearic acids) (*Y*_*6*_), Table 10 lists the percentage MUFA (OA) (*Y*_*7*_) and Table 11 lists the percentage PUFA (LA) (*Y*_*8*_).

The fitted models for ANOVA are summarized in Table 12. Examinations of the two models with an *F*-test and *T*-test indicate a non-significant lack-of-fit at *p* > 0.05. The regression coefficients (*R*^*2*^) of the yield% of solid UCF (*Y*_*5*_), percentage SFAs (palmitic and stearic acids) (*Y*_*6*_), percentage MUFA (OA) (*Y*_*7*_) and percentage PUFA (LA) (*Y*_*8*_) were 0.92, 0.83, 0.61 and 0.80, respectively.

**Table 12:** Analysis of variance (ANOVA) for all the responses of UCF

	**Source**	***Df***	**Sum of squares**	**Mean square**	***F* value**	***P***	
*Y*_*5*_	Model	3	4510.49	1503.50	35.98	< 0.0001	Significant
	Residual	14	484.99	41.79			
	lack-of-fit	12	572.03	47.67	7.36	0.1258	Not significant
	Pure error	2	12.96	6.48			
*Y*_*6*_	Model	3	671.30	223.77	8.39	0.0019	Significant
	Residual	14	373.55	26.68			
	lack-of-fit	12	369.04	30.75	13.65	0.0702	Not significant
	Pure error	2	4.50	2.25			
*Y*_*7*_	Model	9	236.43	26.27	1.43	0.3118	Not significant
	Residual	8	146.77	18.35			
	lack-of-fit	6	139.17	23.19	6.10	0.1475	Not significant
	Pure error	2	7.60	3.80			
*Y*_*8*_	Model	6	940.28	156.71	4.42	0.0163	Significant
	Residual	11	390.43	35.49			
	lack-of-fit	9	348.48	38.72	1.85	0.4004	Not significant
	Pure error	2	41.95	20.98			

Equations 5, 6, 7 and 8 showed that the yield% of solid UCF (*Y*_*5*_), percentage SFAs (palmitic and stearic acids) (*Y*_*6*_), percentage MUFA (OA) (*Y*_*7*_) and percentage PUFA (LA) (*Y*_*8*_) in UCF have a complex relationship with independent variables that encompass both first- and second-order polynomials.

(5) $$\begin{array}{ll} Y_{5}= & +72.14\;+16.64X_{1}-1.83X_{2}+1.65X_{3}\\ & +1.97X_{1}{}^{2}-4.18X_{2}{}^{2}+3.74X_{3}{}^{2}\\ & +2.46X_{1}X_{2}+2.21X_{1}X_{3}-0.81X_{2}X_{3} \end{array}$$

(6) $$\begin{array}{ll} Y_{6}= & +33.80-5.43X_{1}+0.38X_{2}+1.49X_{3}\\ & -1.22X_{1}{}^{2}-0.035X_{2}{}^{2}-3.06X_{3}{}^{2}\\ & -3.96X_{1}X_{2}-0.41X_{1}X_{3}-1.78X_{2}X_{3} \end{array}$$

(7) $$\begin{array}{ll} Y_{7}= & +52.35-14.76X_{1}-1.07X_{2}+0.78X_{3}\\ & -8.33X_{1}{}^{2}+0.71X_{2}{}^{2}+1.58X_{3}{}^{2}\\ & -1.68X_{1}X_{2}+0.39X_{1}X_{3}+0.39X_{2}X_{3} \end{array}$$

(8) $$\begin{array}{ll} Y_{8}= & +13.73+4.16X_{1}+0.83X_{2}-2.07X_{3}\\ & +6.88X_{1}{}^{2}-1.44X_{2}{}^{2}+1.55X_{3}{}^{2}\\ & +5.61X_{1}X_{2}-0.15X_{1}X_{3}+3.74X_{2}X_{3} \end{array}$$

**Figure 9:**
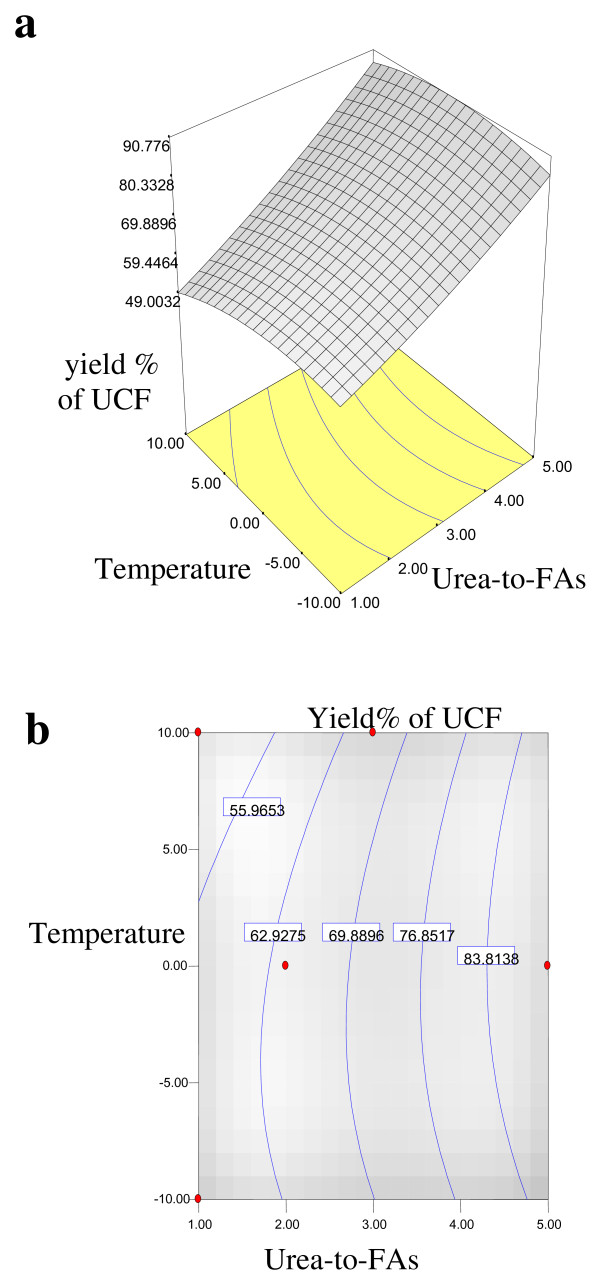
**Response surface (a) and contour plots (b) for the effect of the urea-to-FAs ratio (*X***_***1***_**, w/w) and crystallization temperature (*X***_***2***_**, °C) on the yield% (*Y***_***5***_**) of UCF.**

**Figure 10:**
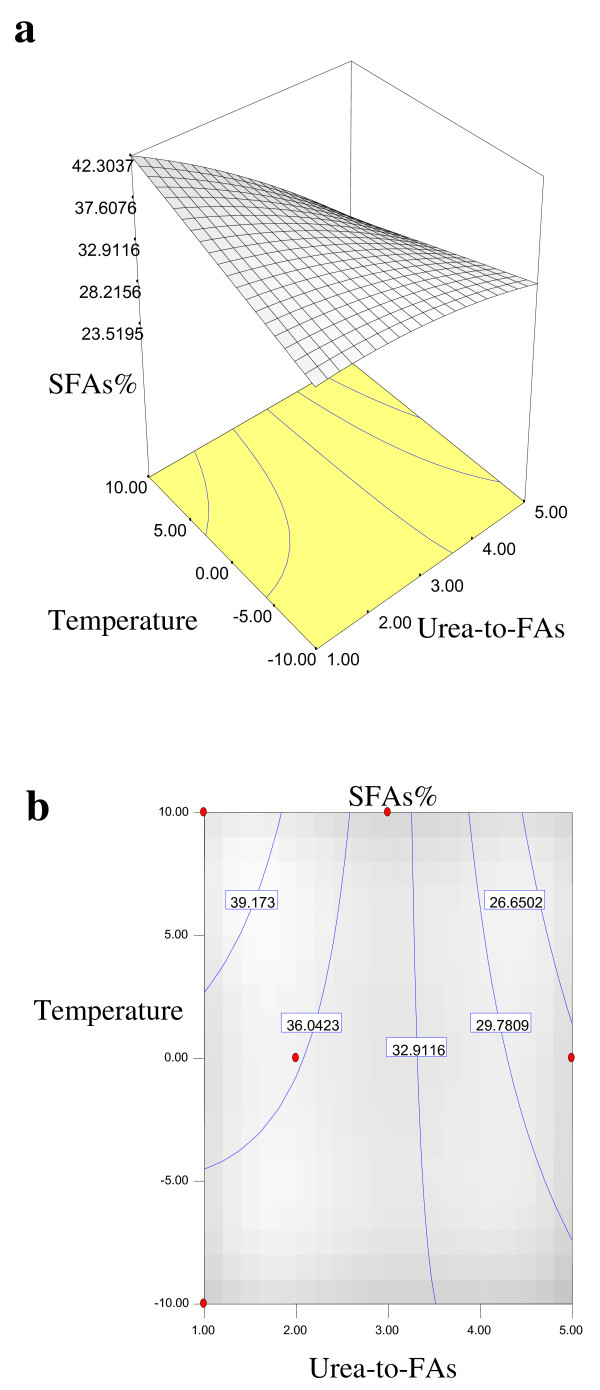
**Response surface (a) and contour plots (b) for the effect of the urea-to-FAs ratio (*X***_***1***_**, w/w) and crystallization temperature (*X***_***2***_**, °C) on the SFAs% (*Y***_***6***_**) of UCF.**

**Figure 11:**
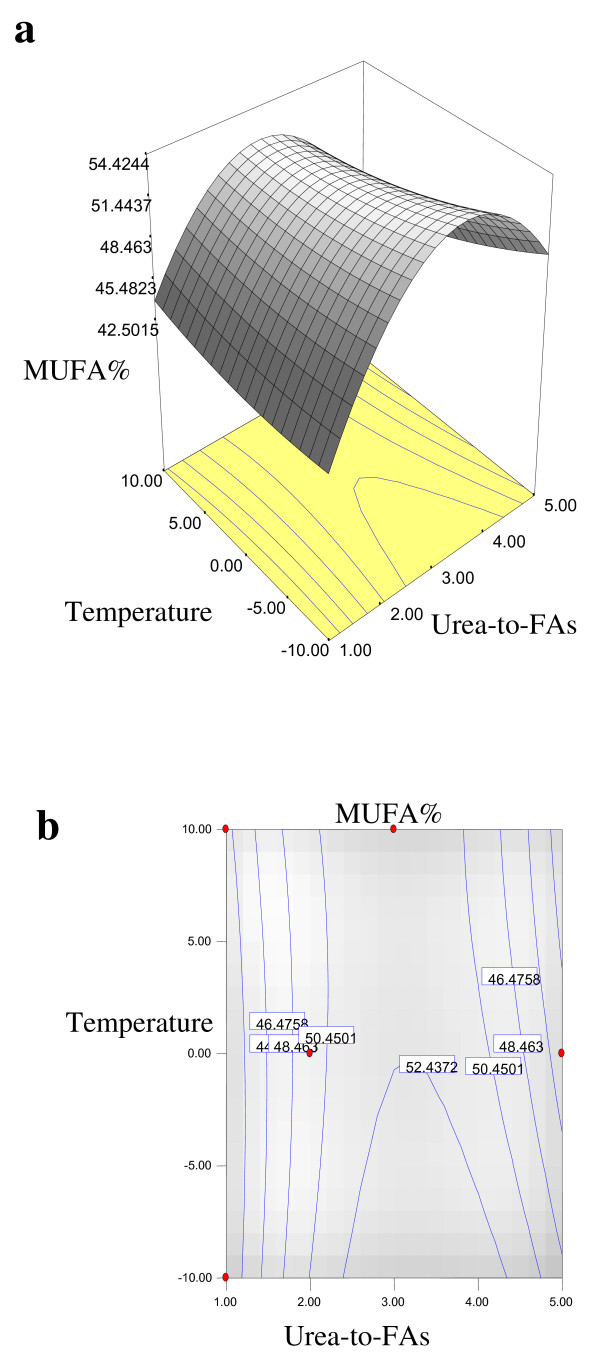
**Response surface (a) and contour plots (b) for the effect of the urea-to-FAs ratio (*X***_***1***_**, w/w) and crystallization temperature (*X***_***2***_**, °C) on the MUFA% (OA) (*Y***_***7***_**) of UCF.**

**Figure 12:**
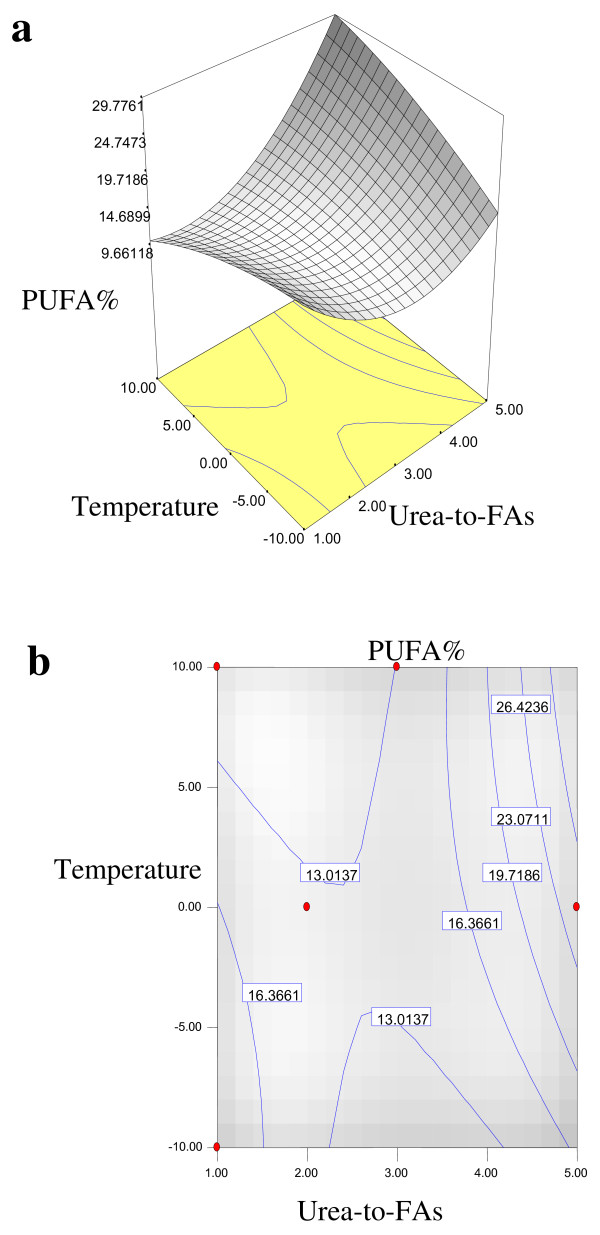
**Response surface (a) and contour plots (b) for the effect of the urea-to-FAs ratio (*X***_***1***_**, w/w) and crystallization temperature (*X***_***2***_**, °C) on the PUFA% (LA) (*Y***_***8***_**) of UCF.**

The response surfaces for the yield% of solid UCF (*Y*_*5*_), percentage SFAs (palmitic and stearic acids) (*Y*_*6*_), percentage MUFA (OA) (*Y*_*7*_) and percentage PUFA (LA) (*Y*_*8*_) in the concentrates are given in Figures 9, 10, 11 and 12, respectively. The contour plots (Figures 9b, 10b, 11b and 12b) show the combination of levels of the urea-to-FAs ratio that can afford the same level of the yield% of solid UCF (*Y*_*5*_), percentage SFAs (palmitic and stearic acids) (*Y*_*6*_), percentage MUFA (OA) (*Y*_*7*_) and percentage PUFA (LA) (*Y*_*8*_).

**Figure 13:**
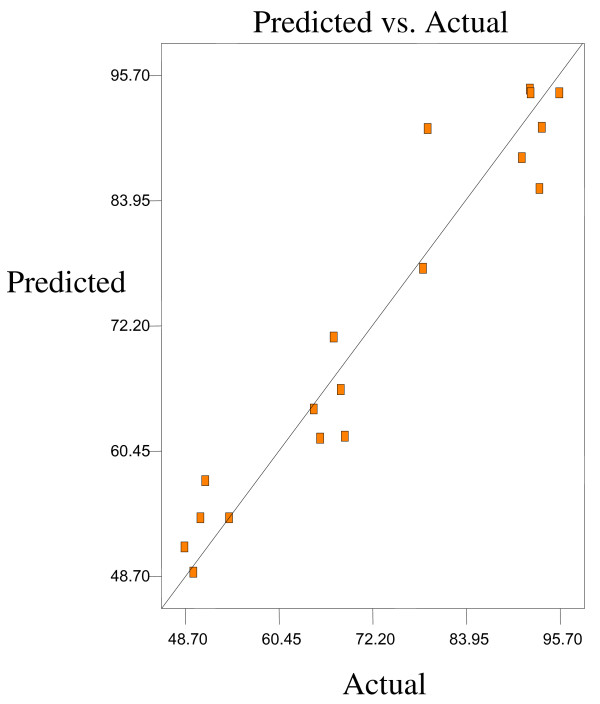
**Predicated vs. actual plot of *Y***_***5***_.

**Figure 14:**
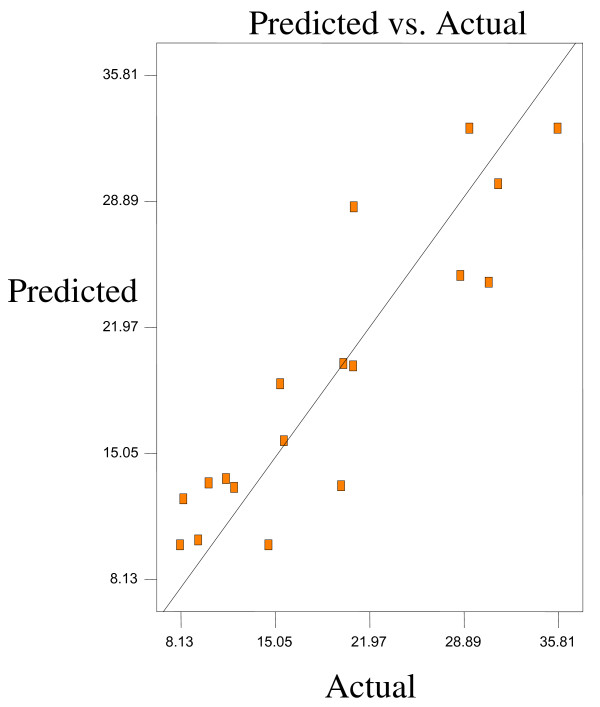
**Predicated vs. actual plot of *Y***_***6***_.

**Figure 15:**
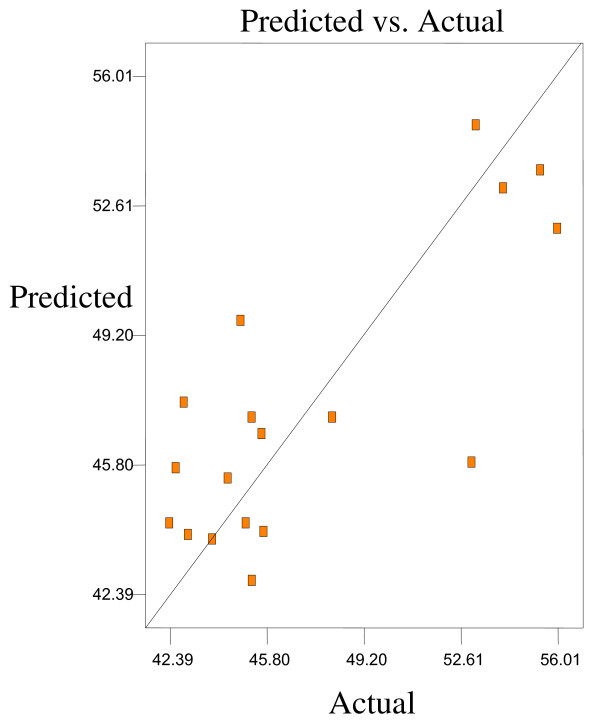
**Predicated vs. actual plot of *Y***_***7***_.

**Figure 16:**
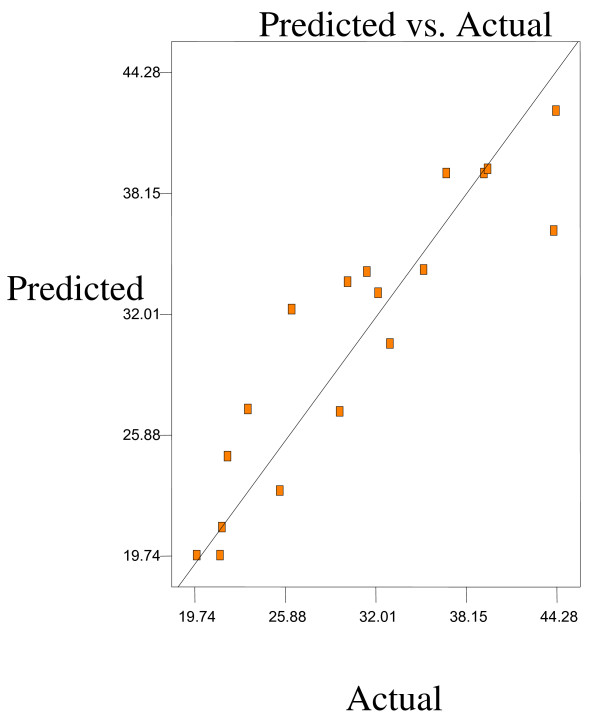
**Predicated vs. actual plot of *Y***_***8***_.

Figures 9, 10, 11 and 12 also represent the Design-Expert plots for all the responses. In the solid UCF, performing the technique using low amount of urea without cooling would give the desired high percentage of SFAs and MUFA (OA) as shown in Figures 10 and 11, respectively. PUFA (LA%) (Figure 12) was lower under these conditions. The observed value was reasonably close to the predicted value as shown in Figures 13, 14, 15 and 16.

## Conclusion

Optimum conditions of the experiment to obtain maximum concentration of PUFA (LA), were predicted at urea-to-FAs ratio (w/w) of 5:1, crystallization temperature of −10°C and 24 h of crystallization time. The final NUCF At this condition was predicted to contain 92.81% of PUFA (LA) with a NUCF yield of 7.8%. The highest percentage MUFA (OA) (56.01%) was observed for sample treated with a urea-to-FAs ratio (w/w) of 3:1 at 10°C for 16 h. The lowest percentage PUFA (LA) (8.13%) was incorporated into the UCF with a urea-to-FAs ratio (w/w) of 1:1 at 10°C for 8 h. All of the above mentioned factors have to be controlled to yield a reasonable amount of product with a desirable purity of FAs.

## Experimental and Methods

### Experimental and Methods

FAs were obtained by the hydrolysis of *J. curcas* seed oil, as carried out by [13]. The separation of PUFA from the hydrolyzed FAs of *J. curcas* seed oil was carried out using the technique describe by [5]. FAs of *J. curcas* seed oil (10 g) were mixed with urea in 95% aqueous ethanol and heated at 60°C with stirring until the mixture turned into a clear homogeneous solution. The ratio of urea-to-FAs (Table 13) was changed by using different amounts of urea (1–5 g). Initially, the urea-FAs adduct was allowed to crystallize at room temperature but lower temperatures were maintained for different periods to allow further crystallization. The crystals formed (UCF) were separated from the liquid (NUCF) by fast filtration. The liquid (NUCF) was diluted with an equal volume of water and acidified to pH 2–3 with 6 N HCl; an equal volume of petroleum ether was subsequently added and the FAs were extracted. The non-aqueous phase (top phase), containing liberated FAs, was separated from the aqueous layer containing urea by filtration. The petroleum ether layer was washed with 5% NaCl solution to remove any remaining urea. The petroleum ether fraction was dried over anhydrous Na_2_SO_4_. The solvent was removed using a rotary evaporator with water bath temperature at 65°C and the FAs converted to FAME for gas chromatography (GC) analysis according to [6]. FAs were analyzed with Shimadzu GC-17A with a BPX70 column (30 m × 0.25 mm × 0.25 μm). Injection and detection (FID) temperatures were set at 260°C and 280°C, respectively and nitrogen was used as the carrier gas with flow rate of 0.3 mL/min. The split ratio was 1:39.

**Table 13:** Independent variables and their levels for D-optimal design of the fatty acids separation

**Independent variables**		**Variable levels**		
		**−1**	**0**	**+1**
The urea-to-FAs ratio (w/w) (g/g)	*X*_*1*_	1	3	5
Crystallization temperature (Â°C)	*X*_*2*_	−10	0	10
Crystallization time (h)	*X*_*3*_	8	16	24

### Experimental design and statistical analysis

A three-factor D-optimal design was employed to study the responses, after urea inclusion fractionation. The yield of NUCF *Y*_*1*_ in % by wt], SFAs (palmitic and stearic acids) *Y*_*2*_ in %], MUFA (OA) *Y*_*3*_ in %] and PUFA (LA) *Y*_*4*_ in %] are shown in equations 1, 2, 3 and 4 respectively. The yield of UCF *Y*_*5*_ in %], SFAs (palmitic and stearic acids) *Y*_*6*_ in %], MUFA (OA) *Y*_*7*_ in %] and PUFA (LA) *Y*_*8*_ in %] after urea inclusion fractionation are shown in equations 5, 6, 7 and 8 respectively. An initial screening step was carried out using the technique describe by [5] to select the major response factors and their values.

The independent variables were *X*_*1*_, *X*_*2*_ and *X*_*3*_ representing the urea-to-FAs ratio (w/w), crystallization temperature (°C), and crystallization time (h). The settings for the independent variables were low and high values: urea-to-FAs ratio of 1 and 5; crystallization temperature of −10 and 10 and crystallization time of 8 and 24. Each variable was coded at three levels: -1, 0, and +1. A quadratic polynomial regression model was assumed for predicting individual Y variables. The model proposed for each response of *Y* was:

(9) $$Y=\beta_{0}+\Sigma \beta i\;xi+\Sigma \beta iixi^{2}+\Sigma \;\Sigma \beta ij\;xi\;xj$$

Where *B*_*0*_; *Bi*; *Bii* and *Bij* are constant, linear, square and interaction regression coefficient terms, respectively, and *xi* and *xj* are independent variables. The goodness of fit of the model was evaluated by the coefficient of determination *R*^*2*^ and the analysis of variance (ANOVA).

## Competing interests

The authors declare that they have no competing interests.

## Authors’ contributions

JS developed the concept, analyzed the data and drafted the manuscript. BMA performed the separation method, optimization study and statistical analysis. NS advised on the methods of tests. All authors read and approved the final manuscript.
